# Left paraduodenal hernia: Embryological and radiological findings

**DOI:** 10.4102/sajr.v25i1.1979

**Published:** 2021-02-17

**Authors:** Atish Vanmali, Jaynund Maharajh, Mario Haines

**Affiliations:** 1 Jackpersad and Partners Inc., Private Practice, Durban, South Africa; 2 Department of Radiology, Faculty of Medicine, King Edward VIII Hospital, Durban, South Africa

**Keywords:** internal hernia, paraduodenal hernia, diagnosis, upper abdominal pathology, surgery

## Abstract

Left paraduodenal hernia (PDH), may present as a surgical emergency with an increased risk of strangulation and incarceration. The diagnosis is challenging because of the non-specific presentation. In the absence of common epigastric or upper abdominal pathology and non-resolving symptoms, a high index of suspicion is required to diagnose left PDH. This report describes a case of radiologically diagnosed left paraduodenal hernia and subsequent successful surgery. It also includes a review of midgut embryology, and the anatomy and radiology of left PDH.

## Introduction

Abdominal hernias are categorised into external and internal hernias. External hernia represents herniation of intestinal loops through a defect in the wall of the abdomen or pelvis. An internal hernia is defined by herniation of a viscus through a normal or abnormal peritoneal or mesenteric aperture within the peritoneal cavity. Internal hernias are rarely encountered in general practice, however, on the background of increased liver transplantations and gastric bypass surgery for bariatric treatment, the incidence of internal hernia is becoming more prevalent.^1^ Internal hernia can be asymptomatic or cause significant discomfort, dependent on the duration and reducibility of the hernia. Hence, this entity may present a clinical challenge and imaging, especially if symptomatic, is imperative. Paraduodenal hernias (PDH) are the commonest of the internal hernias and are classified into left and right, with the former being more prevalent.^1^

## Case report

**FIGURE 1:**
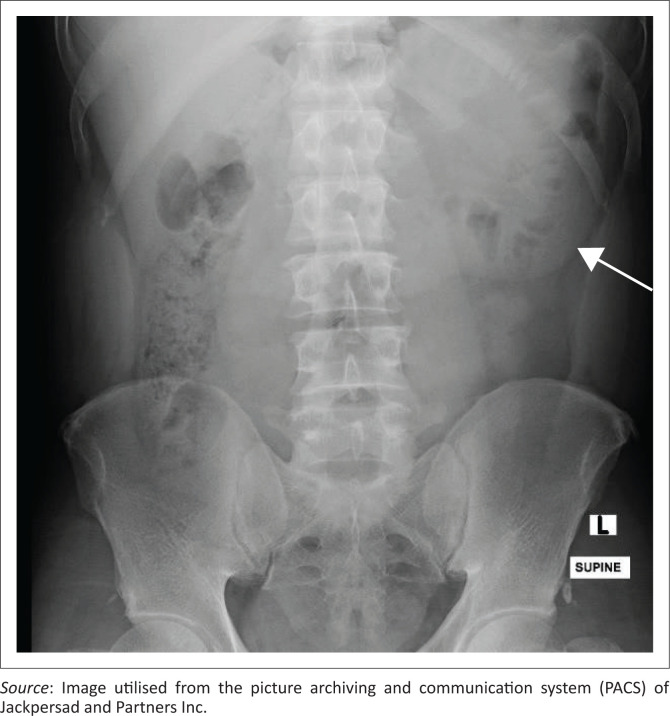
Supine anterior–posterior radiograph of the abdomen, demonstrating a well-circumscribed mass (arrow) projected in the left upper quadrant of the abdomen, representing the left paraduodenal hernia.

The index patient was a 57-year-old male who presented with vague, intermittent, recurrent abdominal pain. The pain evolved into a worsening epigastric pain radiating to the back with associated vomiting. The supine plain radiograph of the abdomen (Figure 1) demonstrated a well-circumscribed mass projected in the left upper quadrant of the abdomen. Free intraperitoneal air was excluded on the erect radiograph (not shown). Additionally, ultrasound excluded cholelithiasis, peripancreatic fluid, a left renal lesion and obstructive uropathy. Markers of infection were minimally increased. Amylase levels were normal.

**FIGURE 2:**
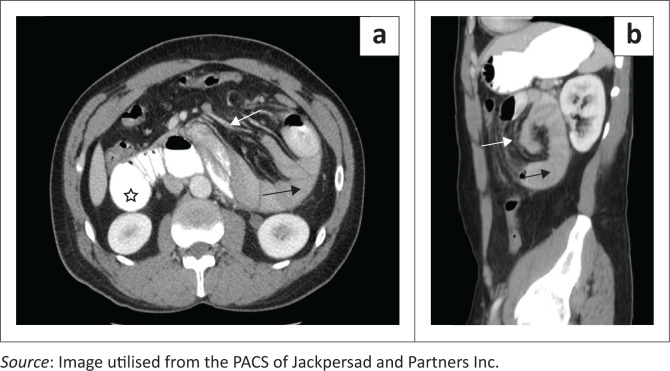
(a) Axial and (b) sagittal portal venous imaging of the abdomen, demonstrating jejunal loops within the left paraduodenal hernia (black arrow) and stretched mesenteric vessels (white arrow) entering the hernial sac. Dilatation of the proximal duodenum (star) is seen with non-visualisation of a normal duodenal jejunal flexure. Note the position of the left paraduodenal hernia, approximating the posterior margin of the stomach.

**FIGURE 3:**
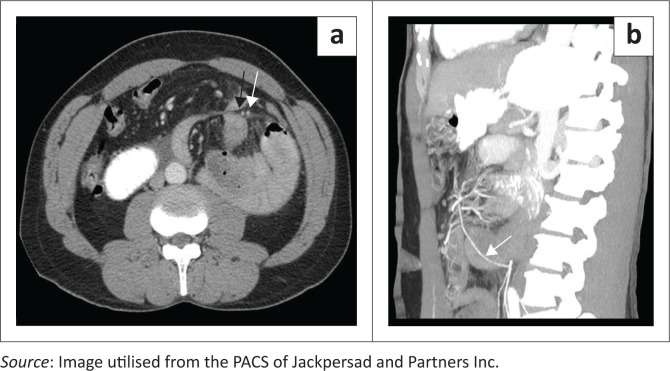
(a) Axial portal venous phase and (b) sagittal reformatted maximum intensity projection images demonstrating the left paraduodenal hernia in relation to the vascular landmarks. Ascending branch of the left colic artery (white arrow) noted on the axial portal venous phase and sagittal reformatted maximum intensity projection image, as well as the inferior mesenteric vein (black arrow) on the axial portal venous phase, approximating the anterior margin of the left paraduodenal hernia.

Given the presence of a left upper quadrant mass on plain radiography that was suboptimally appreciated on ultrasound, computed tomography (CT) was requested. Multiphase contrasted CT imaging was performed with prior administration of oral contrast (Figure 2). A cluster of jejunal bowel loops was noted within a sac in the left upper quadrant of the abdomen. Dilatation of the proximal duodenum and jejunal loops within the sac was evident with loss of the normal duodenal and jejunal configuration. Failure of transit of oral contrast into the terminal ileum was noted. Engorgement of the mesenteric vessels entering the hernial sac was visualised with surrounding fat stranding. There were no features of pneumatosis intestinalis. The inferior mesenteric vein and the ascending branch of the left colic artery were seen along the anterior margin of the hernial sac (Figure 3). These collective findings were in keeping with a left PDH and bowel obstruction.

**FIGURE 4:**
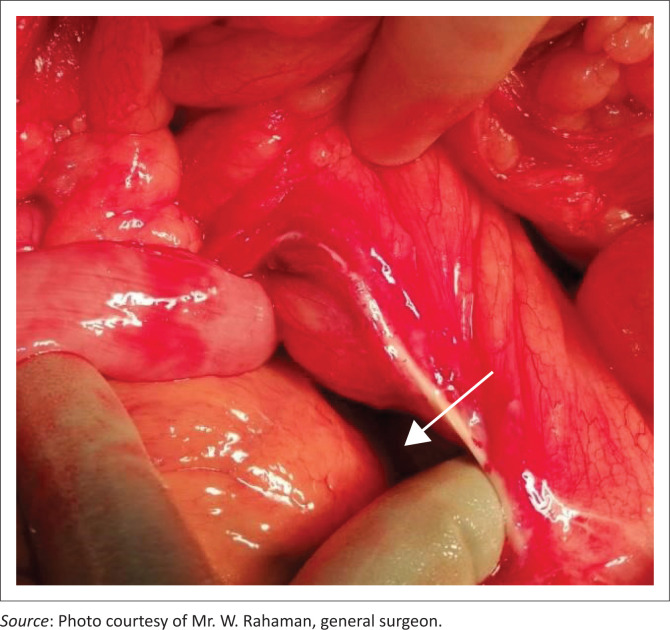
Intraoperative findings demonstrating the congenital defect, fossa of Landzert and the left paraduodenal hernia.

Intraoperative findings (Figure 4) confirmed the congenital defect (white arrow), presence of fossa of Landzert, and jejunal bowel loops within the left PDH. The patient recovered without further complications or recurrence of symptoms.

## Ethical consideration

This article followed all ethical standards for research.Consent was acquired from the patient to include the data and images in the manuscript. Data and images in the manuscript were anonymised.

## Discussion

Left paraduodenal hernia represents an internal abdominal hernia. Internal hernias are defined by the protrusion of a viscus through a normal or abnormal peritoneal or mesenteric aperture within the peritoneal cavity. The orifice may be congenital or acquired, secondary to post-surgical, post-inflammatory or a traumatic defect. Congenital defects include normal apertures, such as foramen of Winslow, and abnormal apertures arising from anomalies of internal rotation and peritoneal attachments.^1^

**FIGURE 5:**
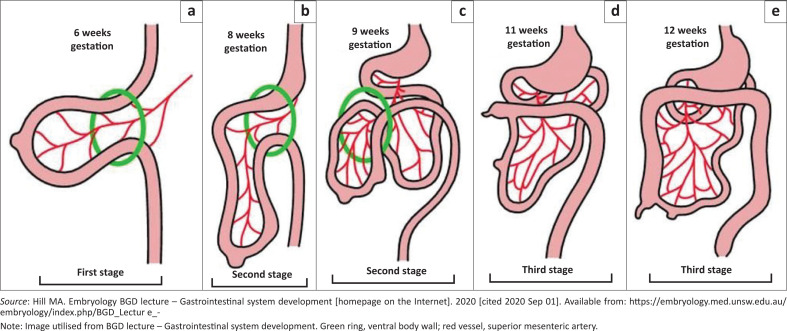
Simplified illustration of midgut reorganisation to establish the normal anatomical position: (a) 6 weeks gestation, (b) 8 weeks gestation, (c) 9 weeks gestation, (d) 11 weeks gestation, (e) 12 weeks gestation.^3^

In understanding the pathogenesis and clinical manifestations of left PDH, it would be imperative to review the embryology of the normal sequence of events relating to the midgut position. The midgut is suspended in the midline by its dorsal mesentery and undergoes a sequential pattern of rotations that is divided into three stages (Figure 5).

This was well described by Bartlett et al.^2^ who reported the first stage beginning at the 5th week in the life of the embryo, in which rapid growth of the abdominal viscera forces a greater component of the midgut external to the abdominal cavity.

In the 10th week of the life of the embryo, the abdominal cavity increases in size and the midgut gradually returns into the abdominal cavity, beginning the second stage of rotations. The midgut has now rotated 90 degrees in a counterclockwise direction on the axis of the superior mesenteric artery, with the pre-arterial segment occupying the right side and the post-arterial segment occupying the left side of the abdominal cavity. The pre-arterial segment rotates an additional 180 degrees counterclockwise, initially posteriorly and subsequently to the left of the superior mesenteric artery, coming to lie to the left of the midline in the abdominal cavity. The post-arterial segment rotates, led by the caecum, which passes counterclockwise, anterior to the superior mesenteric artery into the right upper quadrant of the abdomen, occurring between the 10th and 11th week. The caecum may not completely descend into the right lower quadrant until the end of the 5th month in the life of the embryo.

The third stage represents the fusion of mesenteries and fixation of the midgut. A leaf of the mesentery of the colonic portion of the midgut that has a posterior position, fuses with the peritoneum of the posterior abdominal wall and the space is obliterated. The mesentery of the small bowel is fixed to the posterior abdominal wall and mesentery of the duodenum fuses with the posterior parietal peritoneum.

**FIGURE 6:**
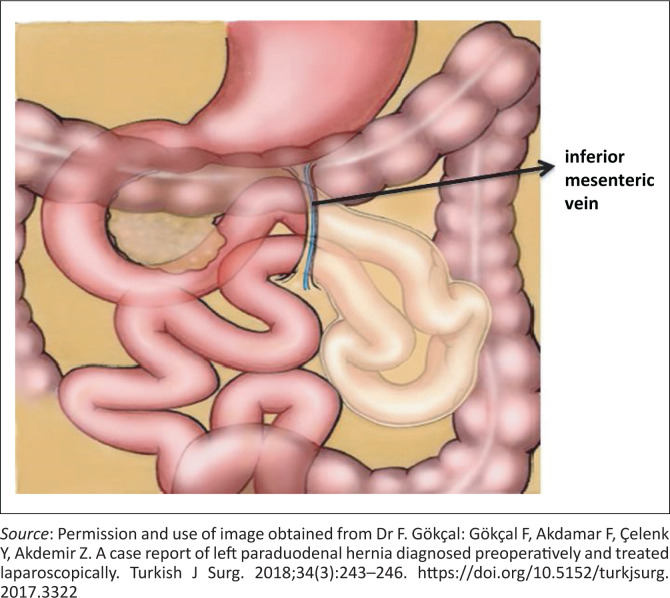
Graphic illustration of a left paraduodenal hernia demonstrating jejunal loops prolapsing into the fossa of Landzert, lateral to ligament of Treitz and posterior to the inferior mesenteric vein and ascending branch of left colic artery.^6^

The mechanism of formation of the left PDH was best described by Callander et al.^4^ Abnormalities in the rotation of the pre-arterial segment in the second stage as it rotates posteriorly and then to the left of the superior mesenteric artery results in a left PDH. During this process, the bowel invaginates into an unsupported area of the descending mesocolon resulting in the anterior margin being formed by the ascending branch of the left colic artery and the inferior mesenteric vein. The small bowel comes to lie in a sac, lined by peritoneum, posterior to the mesentery of the descending colon. The terminal ileum enters the peritoneal cavity through the neck of the sac to reach the caecum (Figure 6).

Treitz has dictated three prereqsuisites for the occurrence of a left PDH: (1) the presence of a fossa (fossa of Landzert), (2) the presence of the inferior mesenteric vein in the neck of the sac and (3) sufficient mobility of the small bowel to allow it into this sac derived from this fossa.^5^

The fossa of Landzert is located to the left of the fourth part of the duodenum, extending posteriorly to the descending mesocolon. The opening is immediately inferior to the duodenojejunal junction and bordered anteriorly by the inferior mesenteric vein and ascending branch of the left colic artery.

Paraduodenal hernias are the most common type of internal hernias, accounting for 50% of cases.^7^ Left PDH is three times more common than right PDH with a male predilection of 3:1.^7^ Left PDHs usually present in the fourth to sixth decade, with a mean age of presentation at 38.5 years.^7^

Paraduodenal hernias often present a clinical challenge. Presentation may range from acute intestinal obstruction, which is the most common presentation, to vague abdominal pain, often relieved by changes in position.^8^ Patients commonly present with postprandial pain typically chronic in nature and dating back to childhood.^1^

Plain radiographs may demonstrate a circumscribed ovoid mass of jejunal loops occupying the left upper quadrant of the abdomen immediately lateral to the fourth component of the duodenum.^8^ Our patient demonstrated a well-circumscribed ‘mass’ projected in the left upper quadrant of the abdomen, with preservation of the left psoas outline and no left lower rib lesions.

The associated risk of strangulation and intestinal infarction of more than 50% over the course of a lifetime, makes it necessary to investigate symptomatic patients. The high rate of mortality associated with these complications justifies the role of CT early in the pre-operative diagnosis of PDH.^9^

The characteristic CT appearance consists of an abnormal cluster or sac-like mass of dilated small bowel loops lying between the stomach and pancreas, to the left of ligament of Treitz. There is usually mass effect that displaces the posterior wall of the stomach, the duodenal flexure inferiorly and the transverse colon inferiorly. The mesenteric vessels supplying the herniated small bowel, appear crowded, engorged and stretched at the entrance of the hernial sac.^10^ The ascending branch of the left colic artery and the inferior mesenteric vein form an important CT landmark along the anterior margin of the hernia.

Our patient presented with vague, intermittent and recurrent abdominal pain that was progressively worsening. The impression of a mass within the left upper quadrant of the abdomen on plain radiography was characterised at CT, which demonstrated pathognomonic features of a left PDH. Urgent surgical intervention confirmed the diagnosis and the patient recovered without complications.

This case report aimed to highlight the importance of plain radiograph interpretation in the background of an internal hernia, which if present, will allude to underlying pathology. Although this may be non-specific, it will support the need for further cross-sectional imaging to delineate the pathology. The case report further describes and diagrammatically illustrates the pertinent aspects of midgut embryology, allowing the radiologist to appreciate the pathogenesis of a left PDH. Identifying the abnormal configuration of the proximal small bowel and associated vascular landmarks assists in the correct interpretation and diagnosis. Internal hernias are rarely seen in clinical practice and it is important for radiologists to familiarise themselves with this concept, as the adverse outcome of an unsuspected or unidentified PDH can result in ischaemia, strangulation and obstruction with mortality between 20% and 50% due to delayed management.^11^

## Conclusion

Left PDH represents a clinically challenging and elusive diagnosis. An understanding of the mesenteric, peritoneal folds, anatomy of the duodenal fossa and embryology of the midgut is important to make the diagnosis. It is imperative for radiologists and surgeons to familiarise themselves with this uncommon condition, especially in patients presenting with non-resolving vague epigastric pain. Plain radiograph may be suggestive, however, CT remains the gold standard for the diagnosis.
